# Yeast Surface Display Platform for Rapid Selection of an Antibody Library via Sequential Counter Antigen Flow Cytometry

**DOI:** 10.3390/antib11040061

**Published:** 2022-09-27

**Authors:** Bhupal Ban, Robert C. Blake, Diane A. Blake

**Affiliations:** 1 Department of Biochemistry and Molecular Biology, Tulane University School of Medicine, New Orleans, LA 70112, USA; 2 Division of Basic Pharmaceutical Sciences, Xavier University of Louisiana, New Orleans, LA 70125, USA

**Keywords:** antibody-based biosensors, yeast surface display, single chain variable fragments (scFv), hexavalent uranium (UO_2_^2+^), affinity maturation, error prone PCR, flow cytometry, equilibrium binding constants

## Abstract

Yeast surface display techniques have been increasingly employed as a tool for both the discovery and affinity maturation of antibodies. In this study, we describe the use of yeast surface display for the selection and affinity maturation of antibodies targeted to small molecules (haptens). In this approach, we coupled 4 to 15 sequential cycles of error-prone PCR to introduce heterogeneity into the sequence of an 12F6 scFv antibody that binds to chelated uranium; the resulting full-length constructs were combined to create a yeast-displayed scFv-library with high diversity. We also developed a stringent selection technique utilizing fluorescence-activated cell sorting; this was based on sequentially dropping the target antigen concentration, while concomitantly increasing the concentration of potential cross-reactive haptens in subsequent selection cycles. As a proof of the efficacy this approach, we confirmed that the antibodies identified via this approach retained binding to the target antigen (UO_2_^2+^ complexed to a chelator), while binding with lesser affinity than the parental scFv to a structurally related haptens (the same chelator complexed to other metal ions). As will be described in this report, these scFv variants perform more efficiently in sensor-based assay than the parental 12F6 antibody. Combining the generation of scFv libraries via error-prone PCR with selection of yeast-displayed antibodies by fluorescence activated cell sorting will provide an efficient new method for the isolation of scFvs and other binding proteins with high affinity and specificity.

## 1. Introduction

The expression and display of proteins on the surface of bacterial and eukaryotic host cells has become increasingly attractive because it provides a rapid and efficient method for the development of novel affinity reagents with increased affinity, specificity, and stability. When a previously isolated monoclonal antibody is used as the starting material, this process is often referred to as “directed evolution” [1]. Directed evolution is a powerful method that involves the random generation of a broad set of protein variants, their expression in a host, and subsequent screening of these variants for those with desired functionalities [2], allowing the improvement of protein properties, the investigation of structure-function relationships, and the study of mechanisms of molecular function. Such variant libraries may be generated via error-prone PCR [3,4,5], DNA shuffling [6], codon-based randomization [5,7] or structurally-guided design methods [8]. Random mutagenesis is distinct from other techniques in that it does not require the researcher to have specific prior knowledge about the 3-dimensional structure of the protein being targeted, thus allowing for the unbiased discovery of novel or beneficial mutations. For this reason, random mutagenesis is especially useful for protein evolution studies.

Yeast surface display has been extensively used to identify and engineer antibody fragments with specific binding activities, including single chain variable fragments (scFv), single domain antibodies (VHH) and non-immunoglobulin scaffold proteins and peptides [9]. This technique allows both identification and isolation of variants with increases in affinity, specificity, and stability [10]. Yeast display has become increasingly accessible and because of its unique advantages, including as host expression with post-translational modification and selection using fluorescence-activated cell sorting (FACS), a useful tool that enables the user to monitor antibody expression, affinity, and selectivity simultaneously [11]. These techniques have been successfully applied to the isolation of new antibodies and for engineering antibodies to alter their affinity and selectivity for a wide variety of antigens, including haptens [11,12,13,14,15].

Antibody based biosensors provide rapid, reliable low-cost detection capability across a broad range of targets but require high stability and a long shelf life in order to be practically useful [15]. Immunoassays (IAs) have become very popular for low-cost, easy-to-use, rapid testing in point-of-care (POC) applications [16]. IA-based devices have been developed for a wide range of analytes of clinical, veterinary, agricultural, and environmental interest, enabling detection of proteins, nucleic acids, drugs, hormones, toxins, viruses, environmental contaminants and bacteria [17,18,19,20,21]. Antibody sensitivity, selectivity, stability, and production yield are major criteria in the development of commercially viable immunosensor-based assays.

Our research group used hybridoma technology to develop a novel anti-uranium monoclonal antibody (clone 12F6) that specifically recognizes UO_2_^2+^ complexed to the chelator, 2,9-dicarboxyl-1,10-phenanthroline (DCP). This chelator was chosen for hybridoma production because it binds to UO_2_^2+^ with an affinity ~5 orders of magnitude greater than that of conventional metal chelators such as ethylenediaminetetraacetic acid (EDTA) [22]. This antibody has subsequently been used for the analysis of uranium in groundwater. Details of the analysis are available in previous work [19,21,23]. Briefly, the 12F6-WT antibody binds to chelated uranium with a very high affinity (K_D_ = 9.1 × 10^–10^ M) and with an 82,000-fold lesser affinity to metal-free chelator (DCP) or other DCP-metal complexes [19]. While this degree of specificity is impressive, the lesser but still significant affinity of 12F6 for metal-free chelator meant that DCP concentrations in field assays had to be carefully controlled at a low concentration (200 nM). Decreasing the DCP concentration in the assays limited the upper range, because when uranium in the sample was >200 nM, there was insufficient chelator to complex all the uranium. When we increased the DCP concentration, we lost sensitivity at lower uranium concentrations. A solution to this problem was to engineer the 12F6 antibody so that it would bind with less affinity to the chelator (DCP) while retaining its affinity for the uranium-DCP complex. The mutant antibody with a higher affinity for UO_2_^2+^-DCP complex was the result of mutation of a lysine in CDR2 of the light chain to an alanine, L^K50A^, (12F6-MT). The re-engineered antibody binds ~2-fold more tightly to UO_2_^2+^-DCP complex and 1/3 less tightly to the metal free DCP [24]. Here, we further improve this mutant 12F6 using methods broadly applicable to any already-useful antibody to improve functions for specific applications. 

**Figure 1:**
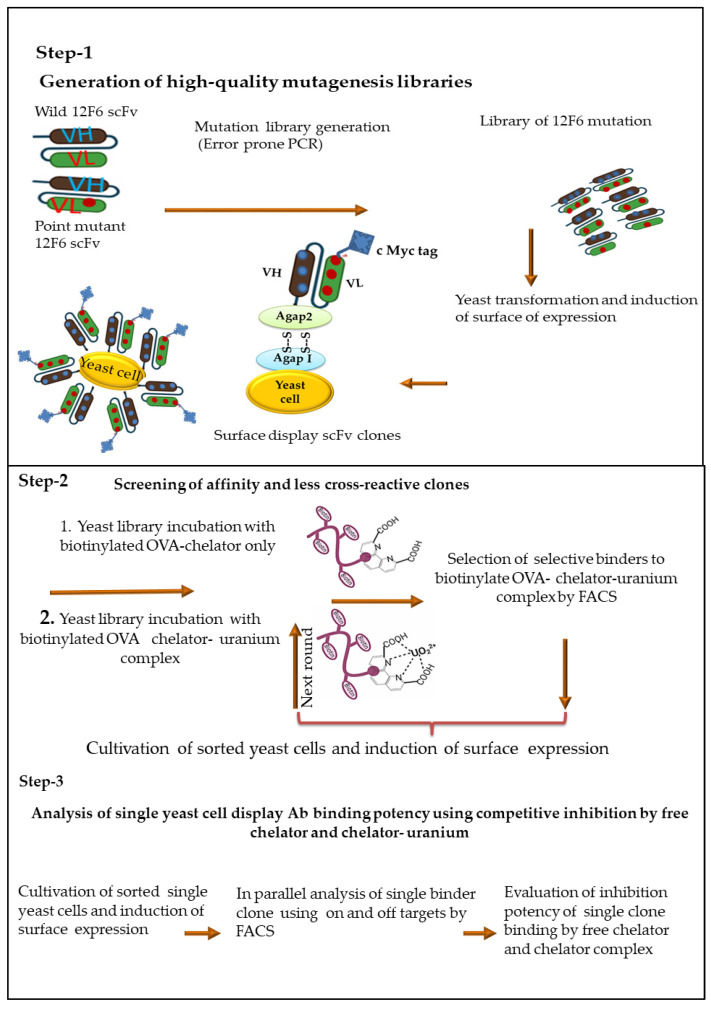
Schematic diagram for mutants’ generation, screening, and selection of selective with desirable affinity antibody using yeast surface display. Step-1 represents the general protocol to generate a quality mutagenesis library using error-prone PCR, and the subsequent packaging of the library into yeast cells. Step-2 represents the screening of specific target antibodies after induction of the surface-displayed scFv library and incubation with the biotinylated protein antigens. Step-3 represents for selection of a single clone yeast surface display antibody with low cross-reactivity to chelator. The fusion protein is tethered to the yeast cell wall via disulfide bridges between the Aga2p protein and the Aga1p protein (which is covalently attached to the cell wall). The fusion protein also contains c-myc affinity tag (EQKLISEEDL) at the C-terminus can be used to monitor the full-length expression of the gene on the yeast surface by flow cytometry. Yeast displays functional antibodies that bind ligand can be identified and isolated by differential fluorescent staining of the antibodies and ligand.

We, therefore, describe an affinity maturation approach which includes (1) mutagenic replication in vitro by a low-fidelity DNA polymerase to introduce large numbers of unbiased newly mutated sequences; and (2) generalized approaches for optimization of both yeast display library generation and screening steps, to achieve rapid isolation of mutants with the desired specificity and affinity (Figure 1). 

## 2. Materials and Methods

### 2.1. Strains, Media, and Antibodies 

Saccharomyces cerevisiae yeast display strain EBY100 (Leu-, Try-phenotype) has genomic insertion of a GAL1-AGA1: URA3 *ura3-52trp1 leu2Δ1 his Δ 200 prp4:HIS2prb1Δ1.6R can1GAL* was maintained in YPD medium [25]. The yeast cells (EBY100) harboring display vector pDNL-6 [24] were grown in SD-CAA minus Trp and Ura medium. For yeast display, the scFv containing yeast cells were grown for 20 h at 18–20 °C in SG/R-CAA medium (identical to SD-CAA medium except glucose was replaced by galactose and raffinose) supplemented with antibiotics [26]. The bacterial strain DH5α *E. coli* DH5α *E. coli* (F-*80dlacZ M15 (lacZYA-argF) U169 recA1 endA1hsdR17 (rk−, mk+) phoAsupE44 -thi-1 gyrA96 relA1*) was used for cloning and propagation of plasmid DNA. The bacterial strain BL21 (DE3) F^−^*ompThsdS*_B_ (r_B_^−^m_B_^−^) *gal dcm* (DE3) was used for the expression of soluble scFv protein under the T7 promoter. The displayed scFv protein was detected by flow cytometry (FACS), using mouse monoclonal IgG that recognizes the c myc tag (9E10) (Thermofisher Scientific, Waltham, MA, USA, CatLog: 980). Phycoerythrin-labeled-goat anti-mouse (H+L) IgG (BioLegend Way, San Diego, CA, USA, CatLog: 405307 and Alexa-633 streptavidin (Thermofisher Scientific, Waltham, MA, USA, CatLog: S21375) were also employed in the FACS analysis.

### 2.2. Preparation of Hapten Protein Conjugates 

#### Preparation of Biotin-Labeled Ovalbumin (OVA)

The biotin-labeled of conjugates DCP-OVA, UO_2_^2+^-DCP-OVA, and OVA were prepared using previously described protocol [11]. These conjugates antigens were used for yeast surface display and ELISA analysis. The biotin-labeled antigens were prepared by activation with NZ-NHS biotinylating kit (Thermo Scientific, Waltham, MA, USA; CatLog: 21435). Briefly, the DCP-OVA conjugate and OVA samples were incubated with biotin in phosphate-buffered saline (PBS) and the low molecular weight reactants were removed using desalting on a disposable PD10 column (Bio-Rad, Hercules, CA, USA) equilibrated with Hepes-buffered saline (HBS, 137 mM NaCl, 10 mM Hepes, pH 7.4). The number of biotin/molecules per protein molecule the using 2-hydroxyazobenzen-4′-carboxylic acid (HABA) system (Pierce Biotechnology, Rockford, IL, USA, CatLog: 28010) and reactions were repeated if the DCP-OVA and control OVA had >10% variance in biotin substitution. A portion of the biotin-labeled DCP-OVA was then incubated with 1 µM UO_2_^2+^ in HBS for 1 h at 37 °C and the unbound free UO_2_^2+^ ion was removed as described above. The protein concentrations of the biotin-labeled UO_2_^2+^-DCP-OVA, DCP-OVA, and OVA concentration were determined using to the BCA method (Bio-Rad).

### 2.3. Construction of Plasmids for Yeast Surface Display (YSD) of Antibody Fragments

In order to engineer more selective anti-uranium antibodies for bio-sensing applications, 12F6 wild type, and crystal structure-based design site-directed single point mutant (L^50K-A^) scFv genes [18], designated 12F6-WT and 12F6-MT, were amplified using a set of primers as described in Supplemental Materials. The amplified PCR products were subcloned into yeast surface display pDNAL-6 vector [24]. In this system, the scFv antibody is expressed as a fusion protein to the mating agglutinin protein Aga2 displayed on the surface of yeast, using the upstream *BssH*II and a downstream *NheI* restriction enzyme that is denoted as pDNL-12F6-WT and pDNL-12F6-MT. These plasmids were confirmed by DNA sequencing. The plasmids pDNL-WT and pDNL-MT were transfected into LiAc treated EBY100 yeast competent cells (Takara Bio USA, Inc, CatLog: ST0029) in the accordingly supplied protocol. The transfected yeast cells were then grown and induced as previously described [25]. In brief, the yeast cell is capable of displaying ~10^5^ scFvs on its cell surface [26,27]. Fluorescence from each cell can be rapidly detected and accurately quantified by flow cytometry. The induced yeast cells displaying wild and mutant of 12F6 scFvs were incubated with soluble antigens and followed by co-staining with anti-c-myc Ab indirectly labeled with goat) anti-mouse (H + L) IgG-phycoerythrin-labeled and streptavidin-Alexa Fluor 633 for epitope binding properties. The affinity of 12F6-WT and 12F6-MT scFvs were monitored by applied primarily 100 nM biotin-labeled-DCP-OVA-UO_2_^2+^ antigen with negative selection antigen 100 nM biotin-labeled-DCP-OVA plus soluble competitor non-labeled metal loaded DCP-OVA-UO_2_^2+^ and metal free DCP-OVA. 

### 2.4. Generation Random Mutagenesis-Based Antibody Fragment Yeast Library 

Beyond standard PCR-based methods for generating diversity, several other methods have been used to create diverse antibody libraries that can be screened by the surface display. Here, we adapted previously reported methods for DNA randomization of the scFv genes via error-prone PCR (e-PCR) [4,5,28] to improve affinity and specificity to meatal loaded chelator DCP-UO_2_^2+^ complex. In brief, the resultant libraries with low mutation rates within variable regions of heavy chain (HC) and light chain (LC) of 12F6 clone genes were generated using set of primers (Supplement Materials). PCR amplification was applied using 12F6-WT and 12F6-MT templates including non-proofreading DNA polymerase operating under sub-optimal conditions utilizing 1 mM Mn^2+^ and 3 mM concentration of Mg^2+^ with a bias nucleotide (1 mM dCTP, 1 mM dTTP, 0.4 mM dGTP, and 0.4 mM dATP) distribution as listed in Supporting Materials (Figure S1). The PCR thermocycle steps were controlled as sequential cycles as 4, 6, 8, 10, and 15 to introduce different magnitudes of mutations per sequence in the library, described in Supplement Material (Figure S1). The mutated 12scFv genes were purified using gel extraction kit (Qiagen CatLog: 28704). PCR products were compatible with yeast display vector; pDNL6, allowing cloning by gap repair [20]. Subsequently, aliquots were either sub-cultured into selective dextrose growth media or plated on selective dextrose growth agar grown at 30 °C. Vector only control transformations were carried out in parallel with the library transformations. Following electroporation, an aliquot of library was serial diluted; decimal serial dilutions (1000 μL) of 10^1^–10^6^ or 10^7^ were prepared from transformed yeast cells. A small aliquot was plated to single cell resolution on dextrose growth agar. The plate was subsequently grown 36 h at 30 °C, resulting in formation of spatially separated colonies. Plate serial dilutions on SD-CAA plates colonies were counted to determine transformation efficiency.

### 2.5. Flow Cytometry Binding Assay for Selection of Specific Yeast Clones against Metal Ion 

Enrichment of the clones that had lower affinity for metal-free DCP while maintaining affinity to the UO_2_^2+^-DCP complex was performed by three rounds of sorting on FACS Aria II (Becton Dickinson) using FACS analysis as described previously [29,30] with modifications. In brief, the induced yeast cells (~2 × 10^7^) were washed in wash buffer I (HBS plus 2 mM EDTA and 0.5% BSA). The mixture was spun down at 13,000× *g* for 30 s and washed twice with wash buffer II (HBS buffer with 0.5% BSA). The induced cells were then incubated with 200 nM biotinylated OVA-DCP-UO_2_^2+^ conjugate with 200 ng/mL concentration of mouse anti-c myc (9E10) antibody in wash buffer II at 25 °C for 1 h. After washing twice with wash buffer II, the sample was incubated with 1:200 dilution of Alexa-633 streptavidin and phycoerythrin-labeled anti-mouse IgG (H+L-IgG-PE) (200 ng/mL) for 1 h at 25 °C. The cells were protected from light during all subsequent steps to avoid photobleaching of the fluorophores. The cells were washed with excess amount of ice-cold wash buffer II and resuspended at a concentration of (about 1 × 10^5^ cells/mL) in HBS buffer with 0.5% BSA to permit slower sampling and more careful separation by FACS. 

### 2.6. Metal Specific scFvs Yeast Library Screening by FACS Sorting 

Typically, the selective gate was set to select the highest 0.1–1% of the yeast population binding to biotinylated OVA-DCP-UO_2_^2+^ conjugate. The sorted cells were grown in SD-CAA medium and induced for scFv surface expression in SG/R-CAA medium before the next round of selection. The concentration of the biotin-labeled ligand (OVA-DCP-UO_2_^2+^) used to interrogate the population of yeast cells was progressively decreased with each round of sorting to increase the binding stringency at the ligand-binding site. In addition, a competitive screening selection again recognition of soluble DCP was included by adding 2 µM of soluble DCP (10-fold molar excess over the biotin-OVA-DCP-UO_2_^2+^) at room temperature. The optimal competition time to achieve maximum fluorescence discrimination depended on k_on_ and k_off_. After 2 initial rounds of sorting, the library was incubated with preloaded biotinylated OVA-DCP-UO_2_^2+^ with increasing concentrations of metal free soluble chelator DCP: 0.3, 1, and 3 µM, respectively. The concentrations of DCP 1 µM and 3 µM were applied for FACS competitive soluble antigens, subsequently, the top 0.5–0.1% concentration-dependent competitive tolerance yeast display scFv clones were sorted. The post-sorted an aliquot yeast cell were plated on SD-CAA agar plates to obtain individual clones for further characterization specific selectivity and affinity against metal loaded-haptent conjugate and mutant clone’s sequences identification.

### 2.7. Evaluation of FACS Sorted Yeast Library Clones against Metal Ion 

In order to identify mutant clones binding more tightly to DCP-UO_2_^2+^ complex compared to metal-free DCP, several single clones were induced as described previously. 139 different mutated clones were tested for specific binding by FACS (LSRII, Becton Dickinson) using biotinylated-OVA-DCP-UO_2_^2+^ alone with large molar excess (5 μM) of unbiotinylated soluble metal-free DCP and 20 nM unbiotinylated soluble DCP-UO_2_^2+^ complex. Data were collected and analyzed based on discrimination of fluorescence intensity at the upper right quadrant (Q2) representing those yeast cells that are displaying on the surface scFv and binding biotinylated OVA-DCP-UO_2_^2+^ complex. Mainly, the individual yeast colonies that were highly tolerance to competitive metal-free DCP, nevertheless, have strong inhibition with metal loaded DCP-UO_2_^2+^ complex were cultivated. The yeast pellet was rinsed with HBS buffer and isolated plasmids DNA using the Zymoprep I kit (Cat Log: D2004) according to the supply method. The extracted plasmids DNA from the post-sorting yeast cells were transformed into *E. coli* DH5α for propagation and isolation plasmids DNA for sequencing.

### 2.8. Soluble scFv Expression and Purification Binding Characterization of Soluble scFvs

Flow cytometry-positive scFvs were cloned into expression vector POE-myc. Details of cloning, bacterial transformation, and induction as described previously [18]. The scFv containing cell pellets were harvested and prepared periplasmic extract as described in detail [18]. The indirect ELISAs against UO_2_^2+^ coated plates as outlined in [22] were performed and the binding properties of the mutants were characterized via competitive ELISA. The parental 12F6-WT and single mutant at position 50 in LC (12F6-MT) scFvs were included on every ELISA plate to provide an internal calibration for results obtained on different plates. Soluble mutants scFvs from periplasmic extract was purified using HisPur Nickle Resin (Life Technology, Grand Island, NY, USA). The periplasmic extract (40 mL) was first incubated with 1 mL resin for an hour with rotation, and then the resin was gravity-packed in a column. The resin column was washed with equilibration buffer (50 mM HBS, 300 mM sodium chloride, 10 mM imidazole, PH 7.4) until the A_280_ of flow through reached a baseline. The scFv was subsequently eluted with 50 mM HBS, 300 mM sodium chloride, 150 mM imidazole, pH 7.4. A few fractions (1 mL each) of eluate were collected to ensure all protein had been eluted, then those fractions with protein were pooled and concentrated using Amicon ultra-15 device 10 kDa cut-off (EMD Millipore, Billerica, MA, USA). The purified scFvs were analyzed by SDS-PAGE and the protein concentrations were determined using the BCA protein assay (Pierce, Rockford, IL, UA). 

### 2.9. Evaluation Selective Clones Binding Affinity and Specificity 

Kinetic exclusion analysis on the KinExA 3000 (Sapidyne Instruments, Boise, ID, USA) was used to determine the equilibrium dissociation constant (K_D_) for the interaction of all mutants scFv antibody of 12F6 with metal-free DCP and the DCP-UO_2_^2+^ complex. The principles and details of these experiments have been reported elsewhere [31,32,33]. Briefly, equilibrium mixtures of the scFv and ligand (DCP-UO_2_^2+)^ or metal-free (DCP) were allowed to flow over a flow/capillary cell that contained beads with immobilized UO_2_^2+^-DCP-OVA. An antibody with no ligand in its binding site was captured by the beads and subsequently quantified with Cy5-labeled 9E10, made in-house using a Cy5 Ab labeling Kit (GE Healthcare, Piscataway, NJ). The signals (delta) at varying ligand concentrations were fit using Slide Write software (Advanced Graphics Software, Carlsbad, CA, USA) and the following binding equation:

y = a0 − (a1 × x)/(a2 + x)

where y is the delta at any given ligand concentration, x is the ligand concentration, a0 is the delta when no ligand was present (y-intercept) and all of the active antibodies is available to bind to the beads, a2 is the equilibrium dissociation constant (K_D_), and a1 is the total change in the value of delta as the x goes from zero to infinity (a0 minus a1 represents the background value of delta at infinite x, when all of the antibodies is saturated with ligand and none is available to bind to the ligand immobilized on the beads). 

## 3. Results

### 3.1. Multistep Random Mutagenesis Library of an Anti-Uranium 12F6 scFv

Anti-uranium mouse monoclonal antibody, 12F6, was generated using hybridoma technology as described 22]. The functional variable antibody genes were identified and converted single variable fragment chain (scFv), thereafter, binding assay to uranium ion were evaluated using previously described method [18]. We performed affinity maturation using a yeast-based scFv expression system using random mutagenesis across the entire V(D)J region by error-prone PCR (epPCR). A separate study showed that antibody libraries with a mutational load of only 1–2 mutations across the entire scFv could effectively be used to isolate high affinity antibody variants. We therefore designed two template-based mutagenesis strategies, one using wildtype 12F6 gene (12F6-WT), the second one crystal structure-based design site-directed single point mutant at position 50 in the light chain (L^50K-A^ 12F6-MT) by epPCR variegation. To determine the efficacy of epPCR mutagenesis, we performed bacterial colonies sequencing to characterize the mutagenized libraries and observed an average of nucleotide codon changing mutations over the length of the scFvs. We determined spontaneously multiple rate mutations including stop codon, insertion, and deletion. Gel electrophoresis performed following different cycles of random mutagenesis showed the expected approximately 750 bp scFv size, as shown in Supplement Figure S1. The distribution of mutations was consistent across the entire scFv with no noticeable bias between the framework regions (FRs) and complementary determining regions (CDRs). We also found that if the epPCR was repeated multiple cycles on the same sample, the number of mutations could reproducibly be doubled or tripled, concomitant with an increase in the introduction of stop codons in the antibody genes, respectively. This mutation not only bound more tightly to UO_2_^2+^-DCP but also was able to discriminate more effectively between metal loaded and metal free chelator.

### 3.2. Generation and Validation of scFvs Display Yeast Cells by FACS Analysis

**Figure 2:**
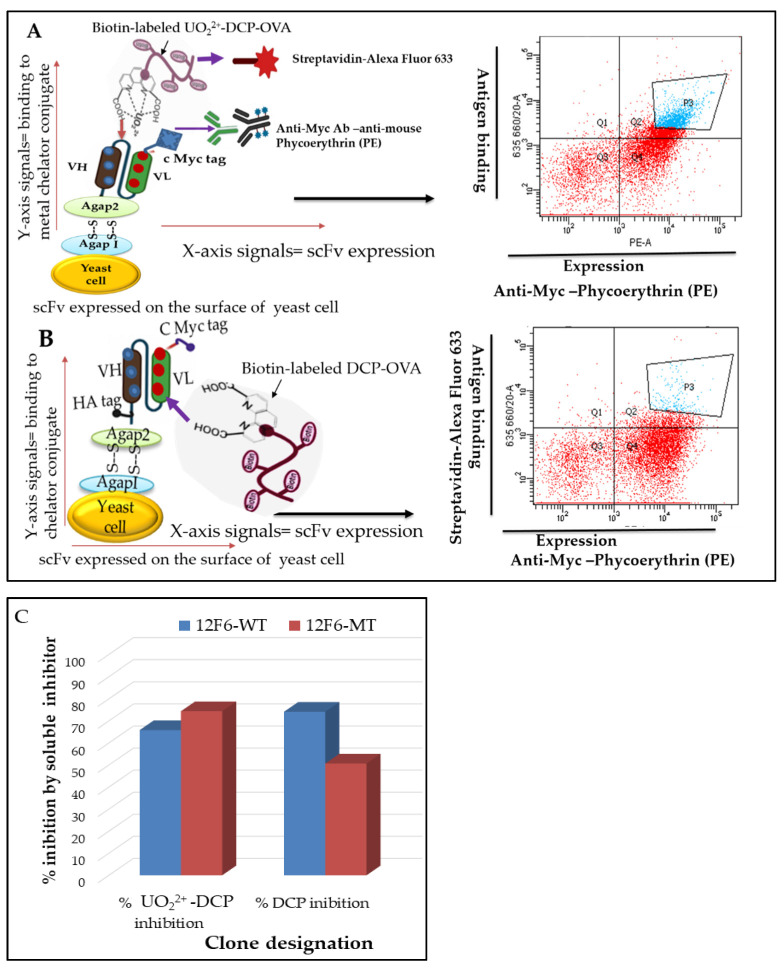
The flow cytometry-based method for assessing induced yeast surface display scFv activities and assessed competitive inhibition by free chelator. In 12F6Ab binding to uranium-chelator assay, positive results occur when binding of biotinylated antigen to induced yeast cells. In the epitope binding assay, a positive result is detected when distinct epitopes are bound by distinct yeast-bound induced scFv, resulting in an scFv antibody detectable by appropriate monoclonal antibodies (mAbs) with phycoerythrin (mAb-PE) and Alexa-635 streptavidin. Light scatter and fluorescence properties of yeast populations aimed for FACS analysis of induced scFv yeast cells; (**A**) Cells were incubated with biotin-labeled DCP-OVA with uranium loaded. (**B**) Cells were incubated with biotin-labeled DCP-OVA without uranium loaded. (**C**) Cells incubated with biotin-labeled 100 nM OVA-DCP-UO_2_^2+^ plus DCP-UO_2_^2+^ (20 nM) and DCP-OVA (5 µM) in buffer, repressively.

To address the complicating selectivity and affinity factors against the small molecule, we used yeast scFv surface display and fluorescence-activated cell sorting (FACS) as illustrated in Figure 2. Considering our aim to improve the affinity and selectivity, we generated yeast scFvs surface display 12F6-WT and 12F6-MT and characterized specific binding against small-molecule. Figure 2A FACS data indicated that 12F6-WT and 12F6-MT scFvs surface display were folded properly, and selectively binding to target antigen metal loaded chelator (DCP-UO_2_^2+^) compared to metal free chelator (DCP) as shown in Figure 2B. The expression level of scFv was monitored by the signal from the myc-tag and is shown on the *X* axis of the flow plot, while the binding capacity of expressed scFv to metal loaded chelator (DCP-UO_2_^2+^) conjugates was detected by the signal from the biotinylated antigen and is shown on the *Y* axis of flow plot. A competitive cell analysis was then performed, yeast pools incubated with soluble competitors; 5 µM DCP metal free chelator (50-fold higher) and 20 nM DCP-UO_2_^2+^ metal loaded-chelator complex (5-fold lower concentration), in the presence of 100 nM biotin-labeled-OVA-DCP-UO_2_^2+^ as shown in Figure 2C. Since the cell population was split after induction of yeast cell surface scFvs, there was no change in the expression level of scFv fragments and the *X* axis signal should remain the same for the 12F6-WT groups. However, those yeast cell-surface antibodies that recognized soluble metal loaded-chelator complex would be competitively inhibited from binding to the biotinylated metal-chelator complex-protein conjugate, and cells carrying antibodies with these binding characteristics should show a decreased signal on the *Y* axis of the flow plot.

### 3.3. Specificity and Affinity Enrichment of Yeast-Displayed Mutated scFv Libraries 

**Figure 3:**
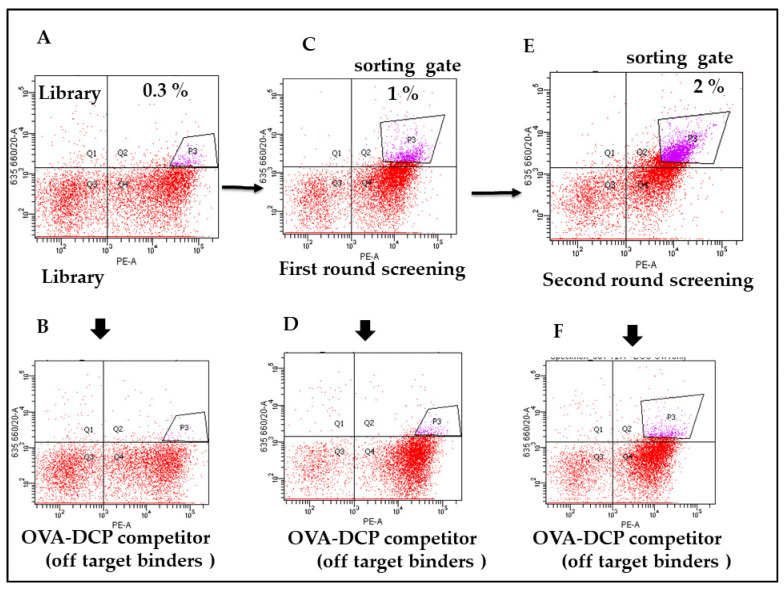

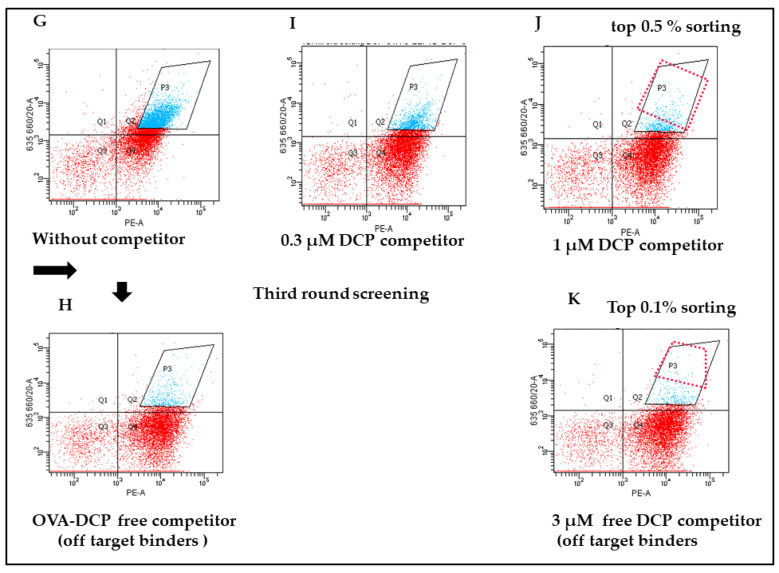
Flow cytometry profiles for parental scFv yeast clones, epPCR, mutagenized libraries, and post-sort libraries. Each row from left to right represents the following samples: parental strain, one, two, or three times sorted libraries, all stained with the relevant soluble antigen. Panel A shows the induced yeast cells population after cloning the 12F6 e-PCR library was incubated at 100 nM biotin-labeled OVA-DCP-UO_2_^2+^. The upper right quadrant (Q2) represents the yeast population that was displaying scFvs on the surface and binding to antigen conations, 0.3% of high binder cells as indicated in (**A**) sort gate were sorted compared to background cells binding as indicated in (**B**). (**C**) represents yeast induced cells were incubated 50 nM biotin-labeled OVA-DCP-UO_2_^2+^, 2% of high binder cells sort gate were sorted as first-round screening. (**E**) represents induced yeast cells were incubated 20 nM biotin-labeled OVA-DCP-UO_2_^2+^, 1% of high binder cells sort gate were sorted. The top binding 1% of yeast cells were sorted from Figure 4C and collected for further induction of surface expression yeast cells binding antigen. (**G**–**K**) represent the yeast cells were that binding with 20 nM UO_2_^2+^-DCP-OVA plus the different concentrations of soluble unlabeled DCP-OVA as competitors, respectively. The top binding 0.5% and 0.1% specific yeast cells were sorted for further analysis. Figures represent (**B**,**D**,**F**) show biotin-labeled- DCP-OVA binding clones representing the off-target as background for all sorts of experiments. Panel L indicates the correlation of off-target versus on-target binding yeast populations. The *y*-axis reflects antigen binding, whereas the *x*-axis displays scFv expression through staining of the c-myc tag.

Three rounds of FACS were performed on each sample, and for each round approximately 1000 cells were isolated from a total population of approximately 1 million cells (Figure 3A–K). The applied antigen concentration for each round of FACS were guided by the antigen binding signal of each input library, with the intention to achieve increased signal in each bound without losing too much of the antigen binding cell population. The intension was also to apply similar selective conditions for two libraries to assess how specificity and the affinity of the parental antibody would influence the resulting complexity and binding affinities of the post-sort of population. Hence, for the first round of sorting 100 nM concentration of antigen was used, subsequently, 50 and 20 nM concentrations were used for consecutive rounds. In each round of sorting the top 1–2% of the antigen positive cells were collected. A square gating strategy was applied, in an effort to select for scFvs with greater specificity and most consistent expressors. This ensured homogeneous scFvs expression for consecutive rounds of sorting and selected against potential mutations of the c-myc tag that were introduced during library construction. In addition, we often observed a substantial increase in absolute fluorescence signal either after the first or second round of sorting. However, those cell-surface antibodies that recognized soluble metal loaded-chelator and metal ion free chelator would be competitively inhibited from binding to the biotinylated metal loaded chelated-protein conjugate, and cells carrying antibodies with these binding characteristics should show a decreased signal on the *Y* axis of the flow plot. Thus, by selecting the cell population that shifted down in the presence of soluble competitor (Figure 3J,K), red. The total cells selected in these gates were ~top 0.5% of the total cells in the absence of soluble hapten (Figure 3J) and ~top 0.1% of cells in the presence of higher concentration competitor soluble hapten (Figure 3K), respectively. 

### 3.4. Competitive FACS of Monoclonal Yeast Cells, Sequence Analysis, and Competitive ELISA of Periplasmic Extracts

**Figure 4:**
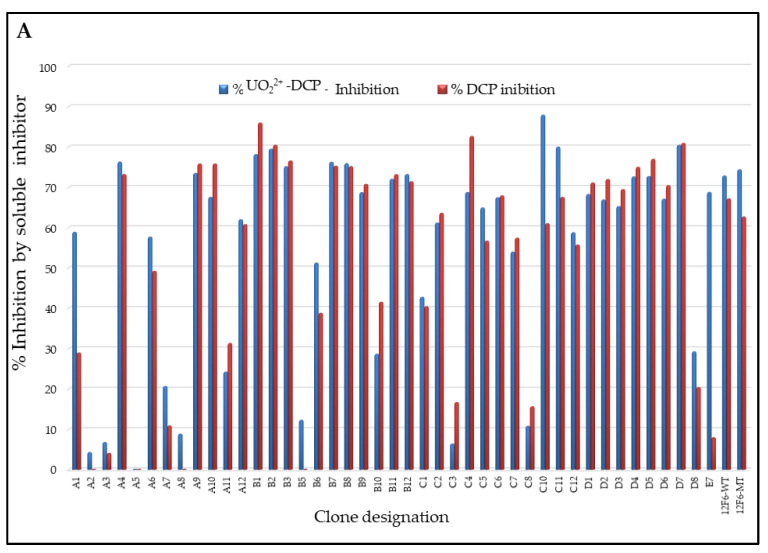

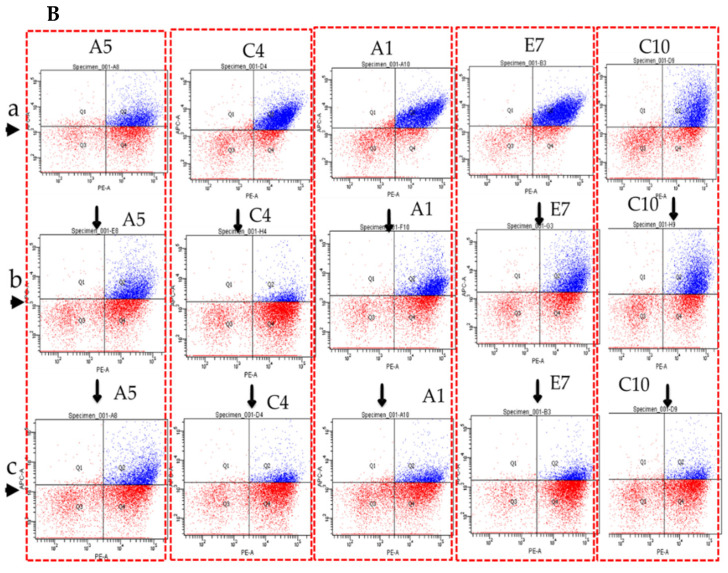
Specificity of selected monoclonal using yeast display. After 3 rounds enrichment using flow cytometry allow the subsequent isolation and testing of single clones. 139 different monoclonals were tested for specific binding to biotin-labeled 50 nM UO_2_^2+^-DCP-OVA plus unlabeled 5 µM DCP-OVA and OVA-DCP-UO_2_^2+^ (20 nM). (**A**): inhibition of binding by DCP and DCP-UO_2_^2+^ selected 12F6 clones. Selective single clone binding to biotin-labeled UO_2_^2+^-DCP-OVA with competitor inhibitors unlabeled DCP and UO_2_^2+^-DCP, respectively. (**B**): Monoclonal FACS of three representative clones is shown in five columns. (Top panel a) Cells were incubated with biotin-labeled 50 nM UO_2_^2+^-DCP-OVA complex; (Middle panel b) Cells were incubated with biotin-labeled 50 nM UO_2_^2+^-DCP-OVA plus DCP-OVA (5 μM) as without metal load in buffer; (Bottom panel c) Cells incubated with biotin-labeled 50 nM OVA-DCP-UO_2_^2+^ plus DCP-UO_2_^2+^ (20 nM) in the buffer.

**Figure 5:**
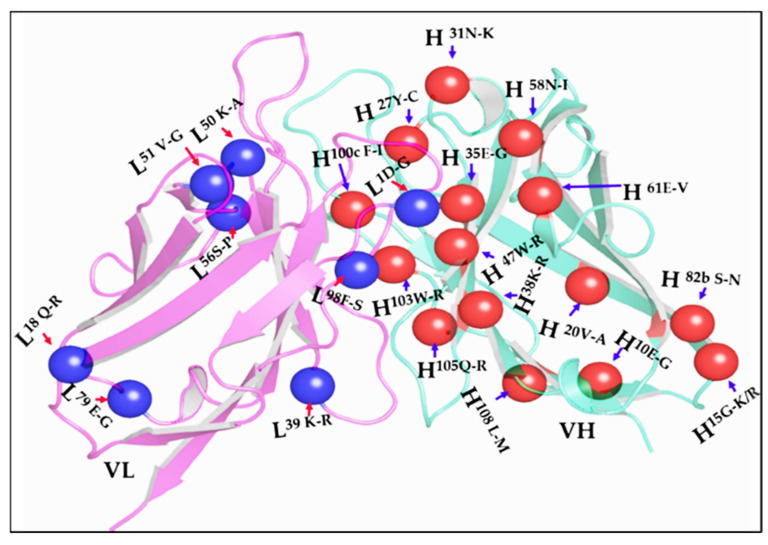
Protein ribbon structure for 12F6 scFv was constructed using PyMOL modeling software. Amino acid residues which were substituted in all variants of 12F6 during the epPCR-based mutagenesis are shown in blue color with a red arrow for the light chain and red color with a blue arrow for the heavy chain. The pink color represents the light chain, and the cyan color represents the heavy chain of antibody ribbon structure.

After the third round of sorting, random 139 FACS sorted single clones were analyzed for their binding selectivity to biotinylated-OVA-DCP-UO_2_^2+^ in the presence competitor soluble DCP or DCP-UO_2_^2+^ antigens. We performed competitive FACS analysis on yeast cell populations derived from these individual clones, the presence of soluble inhibitor by DCP caused a downward shift in the *Y*-axis signal in 109 of the 139 clones (~78%). In addition, we performed competitive FACS on yeast cell populations selected from DCP tolerance in these individual clones, the presence of soluble inhibitor (either DCP or DCP-UO_2_^2+)^ caused a downward shift in the *Y*-axis signal is shown in Figure 4A. The presence of 5 μM DCP and 20 nM DCP-UO_2_^2+^ caused a different downward shift of the signal on the *Y*-axis. The presence of 5 μM DCP or 20 nM DCP-UO_2_^2+^ caused no downward shift of the signal on the *Y* axis when clones were analyzed by competitive FACS; when clones A5, A2, A8, B5, A3, C3, and C8 were analyzed thus, these clones did not seem to be able to distinguish between the DCP and DCP-UO_2_^2+^, respectively. This data appeared to show some preference for hapten-protein conjugate bridge. Of the 30 clones identified by competitive flow cytometry, FACS of five representative example clones (A5, C4, A1, E7, and C10) was shown in Figure 4B. The presence of metal free chelator (5 μM DCP) or metal loaded chelator (20 nM DCP-UO_2_^2+^) caused a similar downward shift of the signal on the *Y* axis. A few clones, A2, A3, and A5 were not seemingly able to distinguish between the DCP and DCP-UO_2_^2+^ complex. Nevertheless, post-sorted clones A1, E7, and C10 appeared to show more selective preference against metal loaded DCP-UO_2_^2+^ complex compared to metal free DCP, since these competitors caused a larger downward shift of the *Y*-axis signal. The C4 clone appeared to show some preference for metal ion free chelator DCP compared to DCP-UO_2_^2+^ and, since this competitor caused a larger downward shift of the *Y*-axis signal. Of these competitive FACS analysis 13 clones were sequenced and subsequently cloned into an expression vector to produce soluble scFv protein, and the crude periplasmic extracts were used to confirm the binding of scFv to soluble OVA-DCP-UO_2_^2+^ ELISA without the avidity or other interferences that might arise from the yeast display system as shown in Supplement Figure S2. Pairs of mutation were found in sorted colonies and mutations residues were tabulated with numbering residues in antibody modeling structure in Figure 5. The alignments of 13 clones’ sequences with the 12F6-WT gene were identified that amino acids substitution mutations occurring often repeatable at positions H^31^, H^35^, H^38^, H^103^, and L^39^, and other single mutations occurred in VH and VL frameworks and including CDRs region. The crystal structure of 12F6-UO_2_^2+^-DCP complex provides an excellent starting point for evaluation mutated residues results. The residues tryptophan-to-arginine at residue H^47W-R^ and H^103W-R^ in FR2 and FR4 have located in the inner core of the binding pocket. Additional, other mutation residues in the core pocket of heavy chain and light chain packing zones such as H^35E-G^ in CDR1, H^100cF-I^ in CDR3, and L^98F-S^ in CDR3 were identified. 

### 3.5. Studies of the Affinity of the 12F6 scFvs Mutants Using KinExA

We hypothesized that FACS with scFv variant libraries might select mutations that improve affinity and selectivity in the scFvs. To investigate, we measured the K_D_ of a selected set of antibodies by equilibrium binding of antigen to soluble scFvs protein expressed in bacterial system. Compared to parental scFv, we calculated binding affinity of selected mutant scFvs to metal loaded and metal free chelator-OVA conjugate by KinExA 3000 (Table 1). The 12F6-WT and 12F6-MT scFvs showed an equilibrium dissociation constant had affinities to metal chelator complex of 7.7 nM, and 4.7 nM, respectively. The binding “window” difference to DCP vs. DCP-UO_2_^2+^ of the 12F6-WT scFv had a 104-fold difference, while 12F6-MT was 225-fold, respectively, as shown in Table 1. To determine whether these affinity-matured antibodies had improved efficacy affinities to DCP vs. DCP-UO_2_^2+^ were similarly measured. The post-sorting 12F6-E7 clone had an affinity of 65 nM to metal loaded DCP-UO_2_^2+^, corresponding to a 185-fold difference between DCP vs. DCP-UO_2_^2+^. Unfortunately, this represents an 8-fold decreased affinity for DCP-UO_2_^2+^ compared with 12F6-WT. The other post-sort enriched scFvs, 12F6-A1, 12F6-A6, 12F6-C10, and 12F6-C5, show 12, 14, 5, and 8-fold reductions in affinity to metal loaded DCP-UO_2_^2+^ complex, respectively, and corresponding decreased affinity 10, 3, 6.5, and 3.8-fold to metal-free DCP, respectively. Of these, compared to the12F6-MT scFv, the post-sort enriched 12F6-E7 scFv had a 10-fold improvement (i.e., 10 times lower affinity) to metal free DCP complex, but unfortunately, this was coupled with binding weaker than the 12F6-MT to metal loaded DCP-UO_2_^2+^ complex. 

## 4. Discussion

Monoclonal antibodies have proven to be versatile affinity reagents for therapeutic and diagnostic applications. Along with the identification of novel lead antibodies, many signs of progress have been made in antibody engineering, in order to improve the antibody developability criteria such as selectivity, specificity, affinity, stability, and efficacy. In particular, the improvement of antibody specificity and affinity are crucial to increase efficacy and enhance therapeutic and diagnostical outcomes. Yeast surface display is the most widely used and powerful approach, taking advantage of an efficient library generation and screening using FACS-based isolation of improved antibody variants [31,33]

In this study, we optimized yeast scFv sorting protocol to evaluate mutagenesis method to attempt to affinity mature antibodies against small molecule. Targeted libraries may be easier to screen but may also miss critical variants in unexpected domains. A larger library of variants requires more effort to screen, but theoretically increases the probability that the library contains an affinity-matured variants. The goal of this experiment was to isolate antibodies that bound to a metal loaded non-covalent complex of UO_2_^2+-^DCP with high affinity and lowering affinity to the unloaded complex of metal-free DCP. Generally, most random V(D)J mutations lead to no change in affinity while most mutations that enhance affinity occur in CDRs, and random mutations in FRs often lead to unstable antibodies. Accordingly, we observed that post-sort epPCR clones contained more mutations in the FRs than CDRs regions. 

For future efforts, there are several process optimizations that could be used to improve results. With a smaller number of samples, we could reasonably sort 5 × 10^7^ cells using FACS or use magnetic-bead-based isolation systems for a larger first round of sorting. Here we used a moderate 20–50 nM antigen concentration and 5 μM competitor off target metal free chelator for all enrichment steps. Thus, especially for the higher affinity antibody-antigen pairs, a further reduction in the antigen staining concentration could more clearly differentiate tighter binders. Alternatively, kinetic screening based on reduced dissociation rate, where the initial stain is followed by a pulse of non-labeled antigen, could identify clones with the desired kinetic parameters.

The results of K_D_ values of 12F6-A6, 12F6-C5, and 12F6-C10 indicate that the change of H^35E-G^ with the dissociation of the side chain presumably decreases the charge density in surrounding residues with changed the affinity to DCP-UO_2_^2+^. It is expected that antigen binding by the CDRs-specific contacts loops of scFv, Fab fragments, therefore; most current antibody engineering strategies focus on antigen-binding CDR loops mutation [34,35] Based on our crystal structural studies of metal ion complex suggested that importantly, the uranyl ion interacting at the antibody binding site is complexed to chelator (DCP), that contains both aromatic nitrogen and carboxylate functional groups and has a binding site that favors larger metal ions. The hydrophobic interface is involved at least in part, binding of either aromatic π-π interaction to metal-free DCP or providing some extensional binding to free chemical residues. The residue heavy chain H^47^ is tryptophane (Trp) in 93% of mouse antibodies [36,37] those important contributions to in pairing the VH-VL interface with L^45^ and V^37^ of VH, all together, form a roughly flat hydrophobic surface that matches well with residues P^44^, F^87^ and F^98^ of VL [38]. The mutated 12F6-D11 clone (H^47W-R^) displayed a 10-fold lower ELISA signal, presumably due to the flexible hydrophobic residues at this position might tend to collapse in an aqueous solution and perhaps the rigidity of the aromatic side chains is required to maintain an open structure (Supplement Figure S2). The VH and VL interface packing pocket is tryptophan residue (H^W103^); (99.3% of mouse VH sequences [39] might have a more structural role in VH and could be important for correct folding or stabilization of the inner core. In addition, the crystal structure of other antibodies complexed with hydrophobic haptens shows a similar “hydrophobic collar” in their binding pocket [40,41]. In fact, 12F6-E7 over 12F6-A1 clones derived scFv antibodies are highly discrimination of binding affinity to metal loaded DCP and metal-free DCP. Interestingly, both mutated had shown the same K_D_ value to DCP-UO_2_^2+^ but differ from unloaded DCP indicating that these residues are not direct interaction with coordinated UO_2_^2+^ free ions as our previous finding L^49Y^ [18], but these residues are directly involved in pairing VH-VL [34]. A number of evidence that involved the residues in core packing at the VH-VL interface, for example, H^39Q-K^ and H^45W-Y/F^ were identified during affinity maturation of HyHEL-10 [36] and H^103W-L^ was identified in 4-4-20 scFv with significant improvement in both affinity and stability [38]. It is intriguing to speculate that the H-CDR3 residues changes are largely responsible for the increase and decrease in specificity for the DCP-UO_2_^2+^ conformation. The selective clone 12F6-E7 binding property provides significant advantages for environmental analysis when compared to the monoclonal and recombinant fragments against chelated UO_2_^2+^ as described previously [18].

The re-engineered antibody 12F6-E7 binds more tightly to DCP-UO_2_^2+^ complex and less tightly to metal free DCP. These differences in affinity more than 1.7-fold the “window” in affinities between the uranium loaded and metal-free chelator to 185-fold. The relatively small “window” in affinity means that the DCP concentration added in our field assays had to relatively low 200 nM. The use of higher chelator concentrations will facilitate the extraction of U(VI) from its natural complexants in groundwater where the environmental protection agency (EPA) action limit for uranium is 126 nM [42]. The high DCP concentrations tolerated by this antibody will also be invaluable in the development of assays for uranium during in situ leaching operations, where uranium complexants are added to groundwater in very high concentrations. 

In conclusion, in this study describes method is very useful for already-useful antibody can be further tailored to improve its function for specific applications. Here, we describe rapid selection procedure against small molecules. We used competitive FACS to isolate antibody populations that could distinguish between chelator and chelator-metal complex, because antibodies for metal selective can serve as markers for environmental uranium contamination. 

## Figures and Tables

**Table 1:** Determination of equilibrium dissociation constants for wild type r12F6scFv and selected mutants of r12F6 scFv. The binding studies were performed as described in [3]. Each experiment was performed in duplicate, and curves drawn through the point were generated using following equation: fraction of occupied sites = [L]/Kd + [L], where [L] is the concentration of soluble UO_2_^2+^-DCP complex and DCP fraction and K_D_ is the equilibrium dissociate constant.

Antibody Variants	Soluble UO_2_^2+^-DCP Complex and DCP Fraction	Equilibrium Dissociation Constant (K_d_, M)	Binding Fold Difference to DCP vs. UO_2_^2+^-DCP Complex
12F6-A1	UO_2_^2+^-DCP	6.1 ± 0.075 × 10^–8^ M	142
12F6-A1	DCP	8.7 ± 0.52 × 10^–6^ M	
12F6-E7	UO_2_^2+^-DCP	6.5 ± 0.08 × 10^–8^ M	185
12F6-E7	DCP	1.2 ± 0.05 × 10^–5^ M	
12F6-C5	UO_2_^2+^-DCP	6.4 ± 0.08 × 10^–8^ M	46
12F6-C5	DCP	3.0 ± 0.05 × 10^–6^ M	
12F6-A6	UO_2_^2+^-DCP	1.1 ± 0.7 × 10^–7^ M	22
12F6-A6	DCP	2.5 ± 0.11 × 10^–6^ M	
12F6-C10	UO_2_^2+^-DCP	3.9 ± 0.08 × 10^–8^ M	134
12F6-C10	DCP	5.2 ± 0.12 × 10^–6^ M	
12F6 -WT	UO_2_^2+^-DCP	7.7 ± 0.9 × 10^–9^ M	104
12F6-WT	DCP	8.0 ± 0.6 × 10^–7^ M	
12F6-MT	UO_2_^2+^-DCP	4.7 ± 0.5 × 10^–9^ M	225
12F6-MT	DCP	1.2 ± 0.09 ×10^–6^ M	

## Data Availability

All related data and methods are presented in this paper and the Supplementary Materials. Additional inquiries should be addressed to the corresponding author.

## Supplementary Materials

The following are available online at https://www.mdpi.com/article/10.3390/antib11040061/s1. References [24,29] are cited in the supplementary materials. Figure S1: Schematic diagram for random mutagenesis by error-prone PCR elements. (A) represents the reagent mixture for e-PCR that mutations are randomly inserted into the DNA sequence under that reduce the fidelity of Taq DNA polymerase. (B) represents thermocycler program for e-PCR. (C) represents e-PCR strategy for dilution and pooling technique for current protocol uses a series of dilution and amplification steps to generate a mutagenic library that contains a range of single-nucleotide point mutation. (D) represents the reaction products analyzed before pooled to create a random library for affinity maturation. Figure S2: Summary of e-PCR mutated residues on variable region of VL and VH of 12F6 mAb. ELISA titter of soluble scFv proteins from e-PCR based mutated variants using crude periplasmic extract. The individuals’ clones were induced under IPTG and preparation of periplasmic extract as detail outlined.
