# Supplementary material for: Yeast Surface Display Platform for Rapid Selection of an Antibody Library via Sequential Counter Antigen Flow Cytometry
Source: Antibodies (Basel). 2022 Sep 27;11(4):61. doi: 10.3390/antib11040061 (PMC9590018; doi:10.3390/antib11040061)
Supplement: Supplementary file 1 [file antibodies-11-00061-s001.zip › antibodies-1710433-supplementary.pdf]

antibodies

MDPI

Supplementary Materials

Yeast Surface Display Platform for Rapid Selection of an Antibody Library Via Sequential Counter Antigen Flow Cytometry

Bhupal Ban [1,†], Robert C. Blake II [2], and Diane A. Blake [1,*]

[1] Department of Biochemistry and Molecular Biology, Tulane University School of Medicine, New Orleans, LA 70112, USA
[2] Division of Basic Pharmaceutical Sciences, Xavier University of Louisiana, New Orleans, LA 70125, USA
* Correspondence: blake@tulane.edu; Tel.: +1-504-988-2478; Fax: +1-504-988-2739
† Current Address: Entrada Therapeutics Inc., 6 Tide Street, Boston, MA 02210, USA.

Citation: Ban, B.; Blake, R.C., II; Blake, D.A. Yeast Surface Display Platform for Rapid Selection of an Antibody Library via Sequential Counter Antigen Flow Cytometry. *Antibodies* **2022**, *11*, 61. https://doi.org/10.3390/antib11040061

Academic Editor: Qi Zhao

Received: 18 April 2022
Accepted: 4 July 2022
Published: 27 September 2022

Publisher's Note: MDPI stays neutral with regard to jurisdictional claims in published maps and institutional affiliations.

Copyright: © 2022 by the authors. Licensee MDPI, Basel, Switzerland. This article is an open access article distributed under the terms and conditions of the Creative Commons Attribution (CC BY) license (https://creativecommons.org/licenses/by/4.0/).

1. Supplemental Materials and Methods

1.1. Media and Agar Plates.

Rich nonselective media yeast extract peptone dextrose (YPD) was prepared in accord with published [1] based for the propagation of EBY100 was prepared using 10 g/L of yeast extract; 20 g/L of peptone; and 20 g/L of dextrose and autoclaved. The YPD plate, 15g/L of agar was added to the above recipe and autoclaved. The selective growth media (SD+CAA) for propagation of EBY100 transformed with pDNL-6. The components are 5g/L of casamino acids (-ade,-ura,-trp), 20g/L of dextrose, 1.7 g/L of yeast nitrogen base without amino acids, 5.3 g/L ammonium sulfate, 10.19 g/L of Na2HPO4.7H20, and 8.56 g/LNa2H2 PO4. H2O was sterilized using filter. For SD-CAA plates, 15g/L of agar was added to the above recipe and autoclaved. SG/R+CCA media for induction of yeast display construction gene using the SD+CAA recipe above substituting D- (+)-galactose and raffinose for dextrose. Antibiotic for SD+CAA and SG/R+CAA cultures were used 50 µg/mL kanamycin and ampicillin after sterilized through filter.

1.2. Preparation PCR mixture and optimization for PCR amplification

The error prone PCR amplification was optimization based on (2) using final concentration 2% DMSO, 3 mM $MgCl_2$, 1 mM dCTP, 1mM dTTP, 0.4 mM dATP, 0.4 mM dGTP using 1 µM forward and reverse primers Primes 12EPFor and 12EPRev were designated to PCR amplify the 12scFv genes from pDNL-12F6WT and pDNL-12F6MT used templates for library construction using 12EPFor -5′-GTTCTGGTGGTGGTGGTTCTGGTGGTGGTGGTTCTGCTAGAGGCGCGCATGCC-3′; and 12EPRev -5′-GAGCACCGTTCAGGTCTTCTTCAGAGATCAGTTTCTGTTCAGCACC-3′ primers using 2ng per reaction target gene in pDNL6 vector as template with 1:10 dilution of Taq polymerase reaction buffer. The PCR mixtures were aliquot 98µl initial tube and 90 µl the rest of 16 individual PCR tubes. Once the PCR programs has reached the annealing temperature, add 1 µl of freshly prepared $MnCl_2$ solution and 1 µl of Taq DNA polymerase to the PCR reaction tube and performed PCR amplification using 100 µl total volume using 3 min extension time for 4 cycles and remove the PCR tube from the thermocycler. Every 4 thermocycles, 10 µl amplified PCR mixture was transformed into new aliquot PCR mixture. This method was repeated cycles 6, 8, 10 and 15 using tubes 6 through 16 to create a mutagenic library by serial dilution amplification as described in Supplement Material (Figure 1S). The PCR products were analyzed using agarose gel and clean up target PCR band using gel purification kit.

1.3. Preparation of yeast competent cells and yeast library

The single clone of EBY100 was inoculate into 3 ml fresh YPD media and grow to stationary phase at 30 °C for overnight, shaking at 220 rpm. Next step, 2 L sterile flask containing 500 mL YPD medium with starting OD600 must be around 0.15–0.2 and

incubate at 30 °C until the OD600 reaches 0.5–0.6 (around 5 h); do not overgrow the culture. The culture samples were sterile centrifuged at 700 g for 10 min at room temperature. Discard the supernatant and resuspend each pellet in 30 ml sterile, deionized water and centrifuged the cells at 700 g for 5 min at room temperature. The supernatant and resuspend each pellet in 6 ml of 1.1× TE/LiAc. The mixture was transferred into high-speed centrifuged tubes and centrifuge at high speed for 30 sec, the supernatant was discarding and added 600 µl of 1.1× TE/LiAc and store the cell on ice. The plasmids were transformed into EBY100 yeast (Trp- phenotype) (Invitrogen) using lithium acetate using Clonetech (ST0029) supplied protocols. Briefly, the yeast population approximately 107 with 0.5 µg plasmid DNA in 400 µL 50% (wt/vol) polyethylene glycol, 50 µL of 1M lithium acetated and 50 µL 10× TE buffer made a mixture PEG/LiAc with 5 µL of ssDNA (10 mg/mL) in a total volume of 555 µL with 50 µL competent cell and the mixtures was incubated at 30 °C for 30 min with gently mixing and added 20 µL DMSO mixed well and incubated 42 °C for 15 min; mix cells every 5 min by gently vertexing. The cells were centrifuged at high speed 15 sec and cells were resuspended in YPD plus and incubated at 40 min for 30 °C with shaking. The cells were centrifuged at high speed for 15 sec and discard the supernatant and resuspend in milliQ sterile water and spread over SD+CAA cultures and grown at 30 °C for 16 h with shaking. The scFv expression was induced by transforming mid log growth cells into SG/R+CAA media with tetracycline and kanamycin to an initial density of 0.5 A600 cells/mL and grown at 20 °C for 24 h with shaking.

1.4. Purification of scFv fragments from periplasm

Periplasmic extracts were prepared from bacterial cells as described previously [2]. Briefly, bacterial cell pellets were resuspended in 1/50 volume of lysis buffer (10 mM HBS (pH 7.4), 1mg/mL lysozyme, and EDTA-free protease inhibitor cocktail). The cells were incubated on ice for 1 h. After incubation, $MgCl_2$ and DNase I were added to final concentrations of 10 mM and 20 µg/mL, respectively. The cells were incubated at room temperature for 30 min and pelleted by centrifugation at 4 °C (20 min, 18000× *g*). The supernatants were collected and stored on ice. Antibody scFv fragments were purified from these periplasmic extracts by immobilized metal affinity chromatography (IMAC). The IMAC resin (HisPurTM Nickel or HisPurTM Cobalt, Fisher Scientific Pittsburgh, PA) was equilibrated in HBS buffer supplemented with 10 mM imidazole and 300 mM NaCl (pH 7.4). Periplasmic extract from 2L of bacterial culture was mixed with 3 mL of equilibrated resin and the samples were mixed end-over-end for 1 h at 4°C. The resin was subsequently packed into a column (0.8 × 4 cm) and washed sequentially with 20 bed volumes of HBS buffer supplemented with 10 mM imidazole and 300 mM NaCl and 5 bed volumes of HBS supplemented with 20 mM imidazole and 300 mM NaCl. The scFv were eluted from the column using an imidazole step gradient from 100 to 250 mM in HBS with 300 mM NaCl. Protein elution was monitored at 280 nm. The collected fractions were analyzed by SDS-PAGE. Protein containing fractions were pooled and activity was assessed using indirect ELISA.

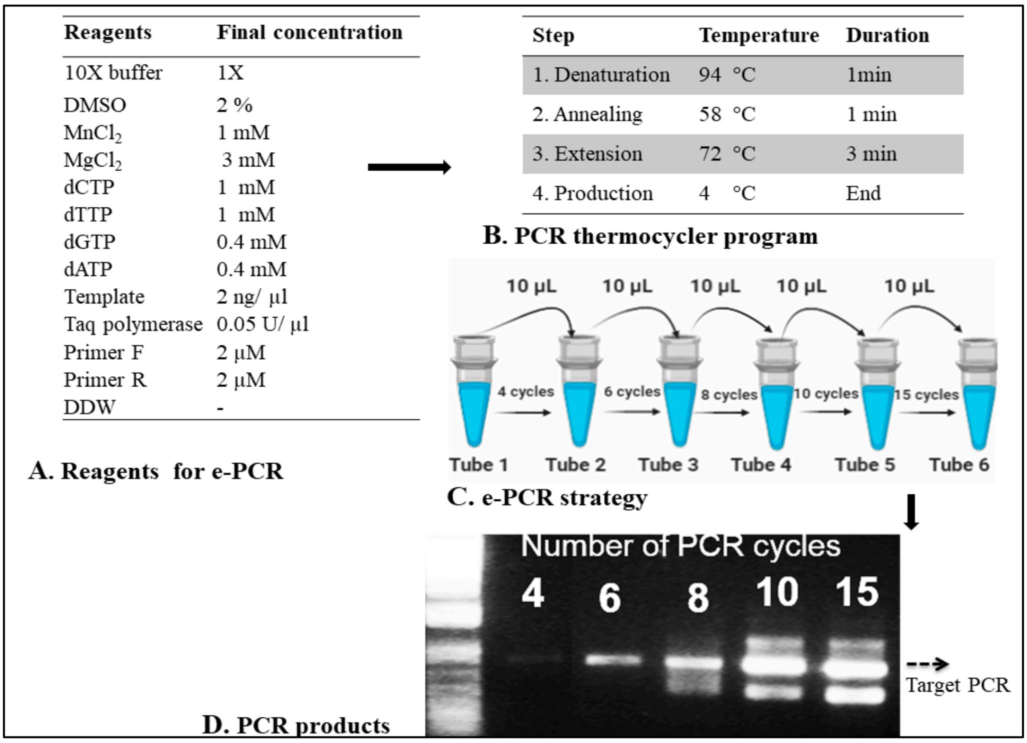

Figure S1. Schematic diagram for random mutagenesis by error-prone PCR elements. Panel A represents the reagent mixture for e-PCR that mutations are randomly inserted into the DNA sequence under that reduce the fidelity of Taq DNA polymerase. Panel B represents thermocycler program for e-PCR. S1C represents e-PCR strategy for dilution and pooling technique for current protocol uses a series of dilution and amplification steps to generate a mutagenic library that contains a range of single-nucleotide point mutation. Panel D represents the reaction products analyzed before pooled to create a random library for affinity maturation.

A.

Denoted mutant clones of 12F6	Mutated residues of Light Chain	Mutated residues of Heavy Chain	Binding study against UO_2^{2+}-DCP-OVA by ELISA (OD 405≅600)
A1	$L^{1D\text{-}G}$, $L^{50K\text{-}A}$	$H^{103W\text{-}R}$	0.989
A3	$L^{50\ K\text{-}A}$	$H^{27Y\text{-}C}$, $H^{35E\text{-}G}$, $H^{105Q\text{-}R}$	0.123
A5		$H^{82b\ S\text{-}N}$	1.452
B3	$L^{79\ E\text{-}G}$	$H^{15G\text{-}K}$, $H^{35E\text{-}G}$	1.785
A6	$L^{56\ S\text{-}P}$	$H^{31N\text{-}K}$, $H^{35E\text{-}G}$, $H^{61E\text{-}V}$	1.479
A10		$H^{31N\text{-}K}$, $H^{35E\text{-}G}$, $H^{61E\text{-}V}$	1.767
B6	$L^{51V\text{-}G}$	$H^{108\ L\text{-}M}$	1.489
C5	$L^{39\ K\text{-}R}$, $L^{50K\text{-}A}$	$H^{10E\text{-}G}$, $H^{35E\text{-}G}$, $H^{58N\text{-}I}$, $H^{100c\ F\text{-}I}$	0.827
C10		$H^{31N\text{-}K}$, $H^{35E\text{-}G}$, $H^{38K\text{-}R}$	1.296
D11	$L^{18\ Q\text{-}R}$, $L^{50K\text{-}A}$	$H^{47W\text{-}R}$	0.27
E4	$L^{98F\text{-}S}$	$H^{15G\text{-}K}$, $H^{20V\text{-}A}$	0.84
E7	$L^{50K\text{-}A}$	$H^{38K\text{-}R}$, $H^{103W\text{-}R}$	0.478
F6	$L^{39K\text{-}R}$	$H^{15G\text{-}R}$	1.41
12MT	$L^{50K\text{-}A}$	-	1.585
12WT	-	-	1.775

B

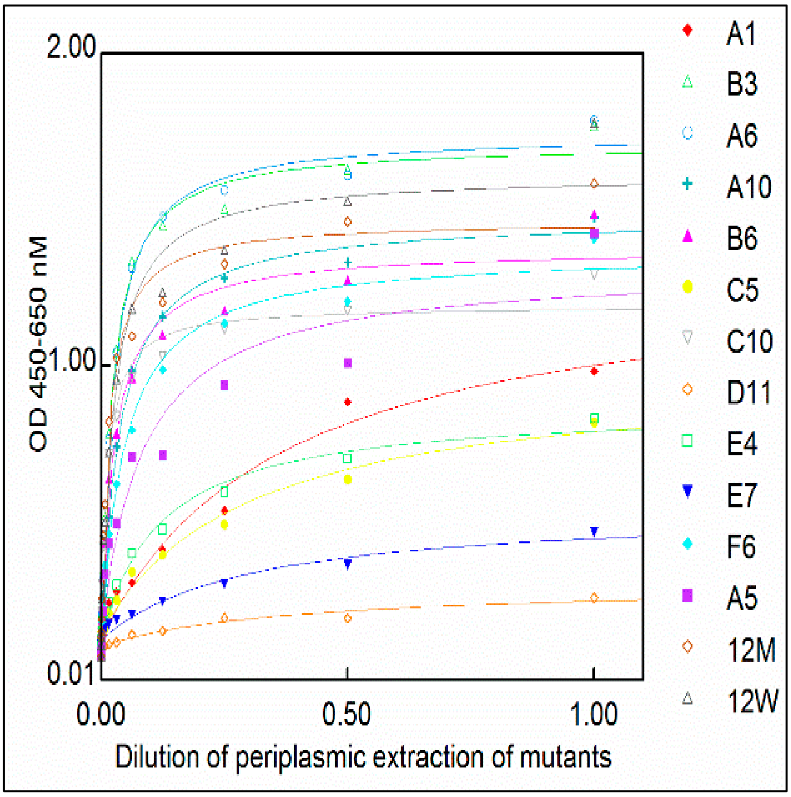

Figure S2. S Summary of e-PCR mutated residues on variable region of VL and VH of 12F6 mAb. ELISA titter of soluble scFv proteins from e-PCR based mutated variants using crude periplasmic extract. The individuals' clones were induced under IPTG and preparation of periplasmic extract as detail outlined.

References

1. Feldhaus, M.; Siegel, R. Flow Cytometric Screening of Yeast Surface Display Libraries. *Methods Mol. Biol. Clifton NJ* **2004**, *263*, 311–332.
2. Zhu, X.; Kriegel, A.M.; Boustany, C.A.; Blake, D.A. Single-Chain Variable Fragment (scFv) Antibodies Optimized for Environmental Analysis of Uranium. *Anal. Chem.* **2011**, *83*, 3717–3724.
